# Cell retention in scalable, perfusion-based mesenchymal stem cell expansion processes: a proof of concept

**DOI:** 10.3389/fbioe.2025.1611703

**Published:** 2025-07-04

**Authors:** Samuel Lukas Schneider, Abiram Gopalakrishnan, Misha Alexander Teale, Regine Eibl

**Affiliations:** Center for Cell Cultivation Techniques, Tissue Engineering and Medical Biology, Institute of Chemistry and Biotechnology, School of Life Sciences and Facility Management, ZHAW Zurich University of Applied Sciences, Wädenswil, Switzerland

**Keywords:** alternating tangential flow filtration (ATF), tangential flow filtration (TFF), microcarrier, hMSC, perfusion, stirred tank bioreactor, single-use technology (SUT)

## Abstract

The production of clinically relevant quantities of human mesenchymal stromal cells (hMSCs) requires scalable and intensified manufacturing processes. For this reason, the applicability of alternating tangential flow filtration (ATF) and tangential flow depth filtration (TFDF) based cell retention systems for hMSC expansion on microcarriers (MCs) in perfusion mode was assessed. The processes were conducted in stirred tank bioreactors at a scale of 1.8 L and compared with repeated-batch cultivations. In the perfusion and repeated-batch control cultivations, competitive viable cell concentrations of ≈2.9 · 10^6^ cells mL^−1^ were reached within a cultivation period of 5–7 days, resulting in an expansion factor of 41–57. The main difference between the operation modi was the aggregation behavior of the MCs. While the median MC aggregate diameter in the repeated-batch cultivation reached 470 μm, the ATF cell retention device constrained aggregate size to a median diameter of 250 µm. In the TFDF cultivation, the shear forces in the recirculation loop stripped most of the hMSCs from the MCs, resulting in the formation of spheroids that continued to proliferate, albeit at a decreased rate. While perfusion operation did not lead to increased productivity in this proof-of-concept study, manual handling and therefore contamination risk were reduced by replacing the repeated-batch process’s daily 80% medium exchanges with automated perfusion operation. Additionally, the ATF system was shown to be useful for medium removal and washing of the MCs prior to adding the harvesting solution, which is highly valuable for cultivations conducted at larger scales. While the feasibility of ATF based cell retention for MC expansion processes could be demonstrated, increased growth area to medium ratios, i.e., higher MC concentrations, still need to be investigated to leverage the full potential of the perfusion process mode.

## Introduction

Human mesenchymal stromal cells (hMSCs), in the past also referred to as mesenchymal stem cells or medicinal signaling cells (Viswanathan et al., 2019), show enormous potential for treating organ damage and degenerative diseases. Their ability for tissue regeneration, immunomodulation and anti-apoptotic activity have as of March 2025 led to over 1,400 clinical trials (https://clinicaltrials.gov/), whereby an exponential increase can be observed (Galderisi, Peluso, and Bernardo, 2021). Applications include but are not limited to osteoarthritis, musculoskeletal defects, ischemic stroke and host versus graft reactions (Teale et al., 2023; Rodríguez-Fuentes et al., 2021). Not only do the hMSCs themselves have great potential for clinical applications, but this also applies to their secretome, mainly through extracellular vesicles (Fan et al., 2020). Their unique cellular functions in combination with their broad range of application make hMSCs ideal candidates for advanced cell therapies.

Since clinical dosages of hMSCs range from 10^6^–10^9^ cells per patient and dose (Kabat et al., 2020), vast numbers of viable and functional hMSCs need to be produced. As scalability and process control are limited in classical 2D cell culture systems like T-flasks and multi-layer-flasks, alternatives have to be evaluated. One of the most promising systems for adherent cell cultivations regarding scalability and process control is the stirred tank reactor (STR) in conjunction with microcarriers (MCs) (X.-Y. Chen et al., 2020; Schirmaier et al., 2014; Lawson et al., 2017). In addition to or as an alternative to scale-up, production processes can be made more efficient through process intensification. In the context of biomanufacturing, this aims to achieve the same or higher productivity using a smaller footprint in a shorter amount of time (Müller et al., 2021). In the comparatively more advanced field of suspension cell processing, e.g., monoclonal antibody production using Chinese hamster ovary cells, perfusion mode operation has established itself as an upstream intensification strategy, allowing for more efficient seed train and production processes. In perfusion mode, “spent” culture medium is continuously removed from the cultivation system through a cell retention device while being replaced with fresh medium. This ensures a stable supply of nutrients while also removing potentially toxic metabolites, making it possible to reach cell densities of >1 ∙ 10^8^ cells mL^−1^, which is substantially higher than what is possible in batch or fed-batch processes (Lavado-García et al., 2022). The current goal is to apply strategies that have shown merit in suspension cell processing to the manufacturing of adherently growing cells. Perfusion operation and associated cell retention devices represent promising technologies in this field since the most commonly used operation mode for stem cell expansions is the “repeated-batch”, in which the culture medium is periodically renewed through partial medium exchanges (MEs), which can be considered a form of discontinuous perfusion. Exchanging or adding medium can promote proliferation as nutrient depletion, growth factor instability and accumulation of metabolites can result in growth inhibition (Pörtner, 2023). However, it should be noted that depletion of accessible growth surface will also result in growth arrest. Therefore, perfusion operation or other medium exchange strategies can only lead to increased productivity if the growth area is not a limiting factor. In cultivation systems in which the growth surface is stationary, e.g., T-flasks and fixed-bed bioreactors, MEs are straight forward. However, for MC based processes where the MCs need to be actively retained in the cultivation system during a ME, they present a serious challenge, particularly at larger cultivation scales exceeding 1 L. Furthermore, to harvest the adherently growing cells from their substrate, the culture medium has to be replaced with a harvesting solution, again requiring a cell retention device.

Different cell retention technologies based on sedimentation (Sion et al., 2021) and dead-end filtration (Dos Santos et al., 2014) have already been used in MC based hMSC expansion processes. However, sedimentation based approaches cannot guarantee 100% cell retention and filtration based approaches can suffer from filter fouling effects (Chotteau, 2015). The most commonly used cell retention devices in suspension cell processing rely on alternating tangential flow filtration (ATF) or tangential flow filtration (TFF), which overcome the limitations of dead end filters (Li et al., 2021; Liang et al., 2024). However, perfusion processes using ATF for hMSC expansion have only been reported once (Cunha et al., 2015a), and TFF was only reported for downstream processing and exosome isolation (Putra et al., 2023; Cunha et al., 2015b).

A well-established bioreactor group that allows for hMSC expansion in perfusion mode is hollow fiber reactors, in which the cells are located within a filter like structure (Russell, Lefavor, and Zubair, 2018; Mizukami et al., 2018). While this approach has the advantage of low hydrodynamic shear stress and a large surface area for cells to grow on, it does not allow direct sampling of the cell culture during the expansion, limiting process insight. Similar to filters, hollow fiber reactors can also suffer from fouling effects during cell expansion. Nevertheless, fully closed and automatable systems like the Quantum bioreactor (Terumo-BCT, Lakewood, CO) have been widely used for clinical hMSC productions (Mennan et al., 2019; Hulme et al., 2023).

Another suitable bioreactor type for MC-based hMSC expansion procedures are wave-mixed bioreactors (da Silva et al., 2019). Due to their more distributed energy dissipation compared to STRs, they offer a more homogeneous shear stress distribution, which can be beneficial for the cultivation of shear sensitive cells (Svay et al., 2020; Löffelholz et al., 2010). However, harvesting relies on temporary high shear conditions to detach the cells from their substrate (Nienow et al., 2014), potentially limiting the application of wave-mixed systems (Tsai and Pacak, 2021). There are wave-mixed bioreactors commercially available with built-in perfusion membranes, facilitating the implementation of perfusion process mode with minimal added complexity and, most importantly, without necessitating external cell retention devices, which could negatively impact cell quality. While perfusion mode processes in wave-mixed bioreactors have not yet been reported for hMSCs, they have been applied to pluripotent stem cell spheroid expansions (Davis et al., 2018).

Large-scale hMSC production is currently not only limited by sub-optimal cell expansion processes but also by the lack of efficient and scalable cell harvest and purification methods (Hassan et al., 2020). As cells are typically the final product, the cell detachment step has a substantial influence on production efficiency and product quality, requiring gentle, yet efficient harvest strategies (Silva Couto et al., 2020). The current state of the art method uses enzymatic treatment combined with mechanical stress. As a first step, the culture medium is removed and, for the harvesting enzymes to work, a washing step is usually required. After the cells have been detached from their substrate, the harvesting solution may be quenched, and the cells need to be separated from the MCs (Nienow et al., 2014). Conducting this process in a timely manner is challenging since shear forces need to be limited while cell losses and aggregation during the multiple liquid removal steps must be minimized (Tsai and Pacak, 2021). Ultimately, cell retention and liquid exchange in a low shear manner is what perfusion devices have been designed for. Therefore, they should be highly applicable to the challenges faced during the harvest of MC based processes, even though higher volumetric fluxes are required at harvest than during perfusion operation.

In this proof-of-concept study, the applicability of Repligen’s ATF and TFF based cell retention devices for perfusion operation and harvesting of MC-based hMSC expansions was evaluated. Repeated-batch cultivations served as controls. The expansion processes were conducted using xeno-free medium in single-use STRs at a working volume of 1.8 L.

## Materials and methods

### Cell line and cultivation conditions

The ASC52telo model cell line (Cat# SCRC-4000) obtained from the American type culture collection (ATCC, Manassas, VA, United States) was used in this study. These hMSCs originate from adipose tissue and were immortalized by transfection with human telomerase reverse transcriptase. The hMSCs were cultivated in the xeno-free Stemline XF MSC medium (Cat# 14371C and 14372C) from Merck (Darmstadt, Germany) supplemented with 2 mM L-alanyl-L-glutamine. To promote cell attachment, all culture flasks were coated using Synthemax II-SC Substrate (Corning Inc., Corning, NY, United States) according to the manufacturer’s instructions, resulting in a coating concentration of 5 μg cm^−2^. For dynamic cultivations, Low Concentration Synthemax II Microcarriers (Cat# 4622, Corning Inc., Corning, NY, United States) were added to the cultivation vessels at a concentration of 10 g L^−1^, resulting in a cultivation area of 3,600 cm^2^ L^−1^. Disposable 125 mL Spinner Flasks (Cat# 3152, Corning Inc., Corning, NY, United States) were used for small-scale comparison cultivation systems. The static culture and the spinner flasks were incubated at 37 °C, 5% CO_2_ and 80% relative humidity. Sub-cultivation was carried out by detaching the cells with 40 μL cm^−2^ TrypLE (Cat# 12563029, Thermo Fisher, Waltham, MA, United States). To remove the harvest enzyme, the detached cells were centrifuged for 3 min at 300 rcf and subsequently resuspended in prewarmed medium.

### Bioreactor setup

**FIGURE 1:**
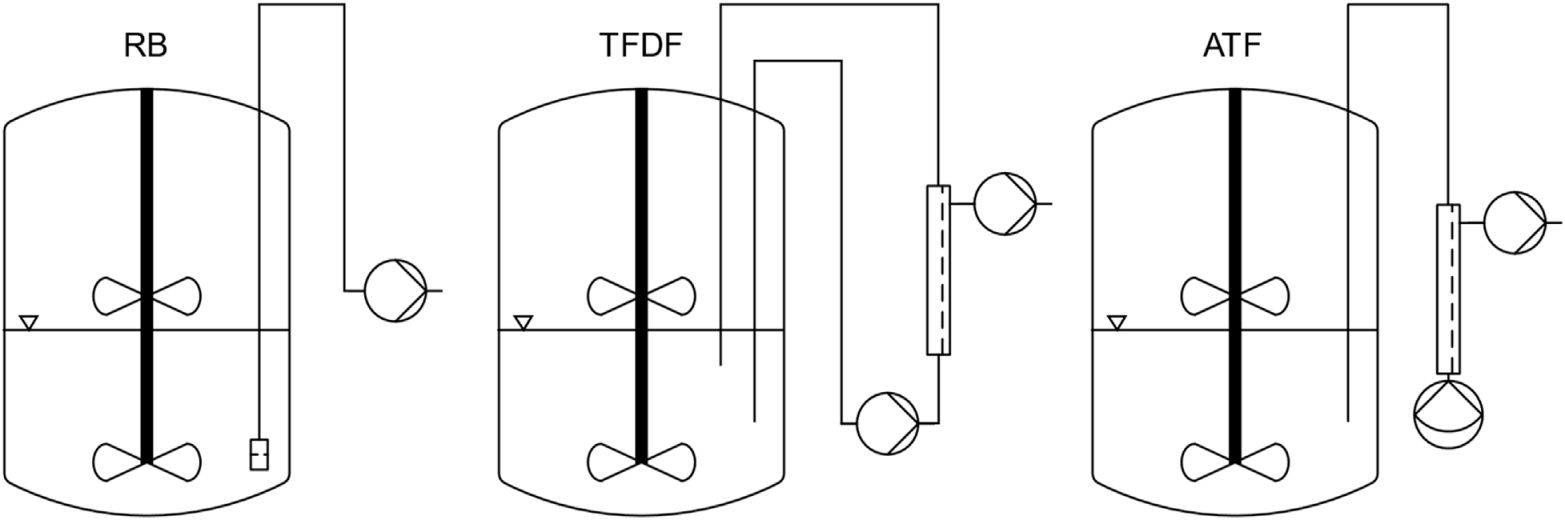
Overview of bioreactor and MC retention device setup for the different process modes.

For all cultivations, the single-use bioreactor BioBLU^®^ 3c (Cat# 1386121000) from Eppendorf (Hamburg, Germany) was used. This bioreactor features two pitched blade impellers, a macrosparger, and an optical pH sensor. Furthermore, the bioreactor was equipped with a temperature- and polarographic dissolved oxygen (DO) probe. A working volume of 1.8 L was chosen, at which only one impeller was submerged. Figure 1 contains an overview of the different microcarrier retention device setups used for the repeated batch, TFDF and ATF cultivations.

For reference repeated-batch cultivations (designated RB1 and RB2), the bioreactor was equipped with a stainless-steel dip-tube (inner diameter: 6.35 mm) covered with an 80 µm woven nylon mesh. This MC retention device made it possible to conduct MEs without losing MCs.

For the TFF perfusion cultivation, designated TFDF, a ProConnex TFDF Flow Path (Cat# STFDFCL15546S) was used in conjunction with a KrosFlo TFDF Lab System (Repligen, Waltham, MA, United States). The installed filter consisted of a single “Tangential Flow Depth Filtration” tube with a lumen diameter of 4 mm and filter area of 30 cm^2^. Two dip tubes (length: 450 and 250 mm, inner diameter: 10 mm) were installed in the bioreactor whereby the flow through the filter module was directed from long to short. In its standard configuration, the TFDF flow path is primed by running the recirculation pump, a PuraLev i100SU (Levitronix, Zürich, Switzerland), at several thousand rpm. To avoid this, a degassing port consisting of a T-connector fitted with an air filter was installed between the return dip tube and the sterile connector. By pulling air through the filter with a syringe, it was possible to prime the flow path without causing excessive shear stress.

The ATF perfusion cultivation was performed using a XCell ATF-2 Single-use Device (Cat# suATF2-S02PES) in conjunction with an XCell Lab System (Repligen, Waltham, MA, United States). The device was installed as recommended by the manufacturer, using a 400 mm long dip tube with an inner diameter of 6.35 mm.

### Agitation rate determination

The repeated-batch expansion procedure was originally developed in spinner flasks and had to be scaled-up to the BioBLU 3c. For this, the *N*_*s1u*_ agitation criterion was chosen, representing the minimal agitation rate at which no stationary MC clusters are formed at the bottom of the bioreactor (Jossen, 2020). The *N*_*s1u*_ determination was performed at 37 °C by adding 18 g MCs and 0.6–1.8 L phosphate buffered saline (PBS) to a fully equipped BioBLU 3c. To allow visual inspection of the bioreactor’s bottom, it was suspended over a 45° tilted mirror and the agitation rate was stepwise increased until no stationary MC clusters were present on the bottom of the reactor for more than one second. As the bioreactor was fitted with pitched blade impellers, the influence of agitation direction (up-pumping vs down-pumping) was assessed. Since the cell retention devices affect the fluid flow in the bioreactor, their influence on the *N*_*s1u*_ was also investigated. With the KrosFlo system, the *N*_*s1u*_ was determined at recirculation flow rates of 0.2 L min^−1^ and 1 L min^−1^ while an ATF flow rate of 0.5 L min^−1^ was tested for the XCell system.

### Inoculum production and inoculation

Inoculum production was carried out in T-flasks and was initiated 7 days prior to the main cultivation start by thawing a cryogenic vial of ASC52telo cells with a passage number of 43. After thawing, the cells were cultured in cell culture flasks and passaged once, 3 days prior to the bioreactor inoculation.

The bioreactor was filled with 1,150 mL medium containing 18 g MCs and equilibrated to the process conditions listed in Table 1 before 100 mL of inoculum was added to reach an initial viable cell density (VCD) of 1.5 · 10^4^ cells cm^−2^ (7.8 · 10^5^ cells mL^−1^). Subsequently, agitation was set to 45 rpm (down-pumping) for 10 min and the first sample was taken. Afterwards, the attachment agitation cycle (3 min on and 87 min off over a period of 24 h) was started. After the 24 h attachment phase, the bioreactor was filled up to its final working volume of 1.8 L and the agitation rate was increased to 57 rpm.

**TABLE 1:** Standard cultivation conditions.

Process parameters and conditions	Setpoint
Agitation rate [rpm] (down pumping)	45 (D0 – D1) 57 (D1 – harvest day)
Agitation cycles during attachment phase	45 rpm for 3 min and 0 rpm for 87 min over 24 h
Temperature [°C]	37
DO [%]	≥40
Inoculation density [cells cm^−2^]	1.5 · 10^4^
MC concentration [g L^−1^]	10
Overlay gassing rate [volume per volume per minute]	0.1
pH [-]	7.2 ± 0.05

The bioreactor was operated using a New Brundswick CelliGen BLU controller in combination with the DASGIP^®^ Control 4.0 software (Eppendorf, Hamburg, Germany). To track the exact working volume throughout the cultivation, the bioreactor was placed on a scale. DO control was realized by sparging O_2_ as soon as the DO value fell below 40%. The pH was controlled at 7.20 ± 0.05 by CO_2_ sparging and sodium carbonate (0.5 M) addition. Temperature was controlled at 37 °C using an electrical heating blanket.

The first 2 days of all cultivations were identical and conducted in batch mode. In the perfusion cultivations, the cell retention devices were primed after 2 days of cultivation, shortly before the perfusion was initialized. In the repeated-batch cultivations, the first ME was performed on day three. After a decline of the specific growth rate was detected, the bioreactors were harvested using prewarmed reagents. In parallel to all main cultivations, a spinner flask repeated-batch cultivation served as a control.

### Repeated-batch cultivations

After an initial batch phase of 3 days, 80% MEs were conducted daily until the bioreactor was harvested. For this, the agitation was stopped to let the MCs sediment for 5 minutes. Afterwards, 80% of the medium was removed through the installed MC retention dip-tube into a sterile 2 L bottle using negative pressure. Subsequently, the same volume of fresh, prewarmed medium was added using overpressure. The removal and addition of medium was controlled based on the bioreactor weight.

To start the harvest procedure, the agitation was stopped and the culture volume was reduced to approximately 200 mL through the MC retention dip tube. For washing, 400 mL of PBS containing 1 mM Ethylenediaminetetraacetic acid (EDTA) was added to the bioreactor and removed after 5 min. Subsequently, the reactor was filled up to 540 mL with PBS containing 1 mM EDTA, before 60 mL of TrypLE Select Enzyme (10X) (Cat# A1217701, Thermo Fisher, Waltham, MA, United States) added, resulting in a TrypLE concentration of ≈10 μL cm^−2^. Cell detachment was conducted under continuous agitation of 57 rpm over 20 min. To quench the TrypLE, 600 mL of previously collected spent medium was added to the bioreactor. Subsequently, the MCs were removed by filtering the suspension through an 80 µm nylon woven mesh. Cryogenic vials of the harvested cells were frozen for later flowcytometry analysis and differentiation assays.

### Perfusion cultivations

To minimize loss of medium and MCs, the cell retention devices were only primed shortly before the perfusion was started on day two. Permeate was removed at a rate corresponding to the target perfusion rate using an external, calibrated peristaltic pump. The KrosFlo’s constant weight diafiltration feature was used to continuously replace the removed medium with fresh one, stored in a 4 °C cooler. The perfusion rate was increased stepwise and corrected during the cultivation leading to a maximum perfusion rate of 1.5 vessel volume per day (vvd) and 1.25 vvd in the TFDF and ATF cultivation, respectively.

For the KrosFlo TFDF System, the recirculation pump was set to 0.4 L min^−1^ on day 2 and perfusion operation was initialized with a perfusion rate of 0.5 vvd. On day 3, the recirculation pump speed was increased to 0.5 L min^−1^. After 7 days of cultivation, the bioreactor was harvested. The planned harvesting procedure was to reduce the volume to 500 mL and include a diafiltration step using the TFDF System at 0.5 L min^−1^ recirculation rate and 50 mL min^−1^ permeate flow. However, this led to blockage of the filter after about 1 L of permeate was collected. For this reason, the recirculation loop was emptied, and the harvest was carried out using the same procedure as in the repeated-batch cultivations.

On day two of the ATF process, the ATF flowrate was set to 0.5 L min^−1^ and perfusion operation was started at 0.5 vvd. The harvest was initiated after 6 days by increasing the ATF flow rate to 1 L min^−1^ and reducing the culture volume to 540 mL within 30 min. Subsequently, a diafiltration step with PBS containing 1 mM EDTA was performed where 1 L buffer was passed through the bioreactor over 25 min. To improve cell recovery, the ATF-2 device was drained prior to adding 60 mL 10× TrypLE, resulting in a TrypLE concentration of ≈10 μL cm^−2^. Subsequently, the same procedure was followed as in the repeated-batch cultivations.

### Sampling procedure

Every 24 h, a sample was drawn from the bioreactor. To ensure homogeneous suspension of the MCs, the agitation rate was increased to 70 rpm for 2 min prior to sampling. Immediately after sampling, a pH measurement was taken and used to correct the online pH, if necessary. For cell density determination, 5 mL sample was centrifuged for 1 min at 200 rcf and the supernatant was replaced with 2 mL prewarmed TrypLE, resulting in a TrypLE concentration of ≈11 μL cm^−2^. After 10 min of incubation at 37 °C, the detached cells were measured using Via1-Cassettes (Cat# 941–0,012) on a NucleoCounter NC-200 (ChemoMetec, Allerod, Denmark), resulting in information about the VCD, total cell density, cell diameter and viability. The previously removed supernatant was used to measure glucose (Glc), lactate (Lac), ammonium (NH_4_^+^), glutamine (Gln) and alanyl-glutamine (AQ) concentrations with a Cedex Bio Analyzer (Roche, Basel, Switzerland). Additionally, 1 mL of the MC suspension was fixed using 10% formalin and stored at 4 °C for later cell distribution analysis. After all samples have been collected, they were washed once with PBS and subsequently permeabilized and stained with 286 nM 4′,6-diamidino-2-phenylindole (DAPI) in PBS containing 3 mL L^−1^ Triton X-100. After the staining, the MCs were washed once with PBS and embedded in 3 g L^−1^ agarose for microscopic imaging. Images were evaluated using a custom MATLAB (The MathWorks Inc., Natick, MA, United States) script which detects cells and MCs based on static brightness thresholds applied to the brightfield and DAPI channel, respectively.

### Cell quality assessment

The cell quality of the harvested cells was assessed based on the minimal criteria published by (Dominici et al., 2006). The presence or absence of the markers listed in Table 2 was measured using a MACSQuant Analyzer 10 flow cytometer (Miltenyi Biotec, Bergisch Gladbach, Germany). All required reagents were obtained from Miltenyi Biotec.

**TABLE 2:** List of analyzed surface markers.

Marker	Type	Conjugated fluorochrome
CD14	Negative	VioBlue
CD19	Negative	VioBlue
CD34	Negative	VioBlue
CD45	Negative	VioBlue
HLA-DR	Negative	VioBlue
CD73	Positive	Allophycocyanin (APC)
CD90	Positive	PE-Vio 770
CD105	Positive	Phycoerythrin (PE)

The flowcytometry data was pre-processed by excluding debris based on the forward and sideward scatter signals, followed by a singlets gate which was based on the forward scatter height to forward scatter area ratio. An event was counted as positive if its fluorescent intensity was higher than 99% of all corresponding isotype control events.

In addition to flow cytometry, the cell quality was assessed by directed differentiation into osteoblasts, adipocytes and chondroblasts using the kits listed in Table 3. The differentiations were carried out according to the manufacturer’s manual. Dyes and staining solutions were purchased from Sigma-Aldrich (St. Louis, MO, United States).

**TABLE 3:** Differentiation media used for trilineage differentiation assay.

Product name	Catalog number	Supplier	Differentiation duration [d]	Staining	Target
Adipogenic Differentiation Medium 2	C-28016	PromoCell (Heidelberg, Germany)	14	Oil Red O	Neutral lipids
ChondroMAX Differentiation Medium	SCM123-100 ML	Sigma-Aldrich (St. Louis, MO, United States)	21	Alcian blue 8GX	Negatively charged glycosaminoglycans
StemMACS OsteoDiff Media	130–091–678	Miltenyi Biotech (Bergisch Gladbach, Germany)	14	Alizarin Red S	Calcium deposits

For the evaluation of adipogenic differentiation, cells were fixed after 14 days using Saccomanno’s fixation solution, followed by a 20 min staining with a 3 g L^−1^ Oil Red O (Cat# O1391) solution, prepared in 0.6 L L^−1^ aqueous isopropanol. Subsequently, the cell layer was washed with 0.6 L L^-1^ isopropanol and staining was evaluated by light microscopy.

Chondrogenic differentiation was evaluated after 21 days by alcian blue staining. For this, the cell aggregates were fixed for 1 h in 10% formalin and washed with PBS and destaining solution before being stained for 1 h. After subsequent destaining, the aggregates were photographed. The destaining solution consisted of 0.5 L L^−1^ ethanol and 0.15 L L^−1^ acetic acid in water, while 10 g L^−1^ alcian blue 8 GX (Cat# 05500) was additionally added to create the staining solution.

Osteogenic differentiation was evaluated after 14 days by fixing the cells with Saccomanno’s fixation solution and staining with Alizarin Red S. The staining solution consisted of an aqueous 20 mg mL^−1^ Alizarin Red S (Cat# A5533) solution which had its pH adjusted to 4.2 with ammonia. After 5 min of staining, the samples were washed several times with deionized water and imaged.

## Results

### Suspension criteria evaluation

To minimize shear stress experienced by cells during cultivation, the agitation rate was set to the *N*_*s1u*_ criterion, which corresponds to the lowest agitation rate at which no stationary MC deposits are formed. The *N*_*s1u*_ was elucidated at different bioreactor filling levels and configurations. Initially, the influence of the agitation direction was assessed without any cell retention system at the targeted cultivation volume of 1.8 L, resulting in a *N*_*s1u*_ of 57 and 95 rpm for down-pumping and up-pumping, respectively. Due to the much lower agitation rate required to reach the *N*_*s1u*_ criterion in the down-pumping mode, only this agitation direction was further investigated. At the inoculation (1.25 L) and harvest filling level (0.6 L), the *N*_*s1u*_ was determined to be 45 and 50 rpm, respectively. At the cultivation volume of 1.8 L, the influence of the cell retention systems on the *N*_*s1u*_ was additionally assessed. It was found that the TFDF system did not noticeably influence the *N*_*s1u*_ at recirculation flow rates of 0.2 and 1.0 L min^−1^. Additionally, sedimentation of MCs in the recirculation loop was not found to be problematic at the tested flow rates.

**FIGURE 2:**
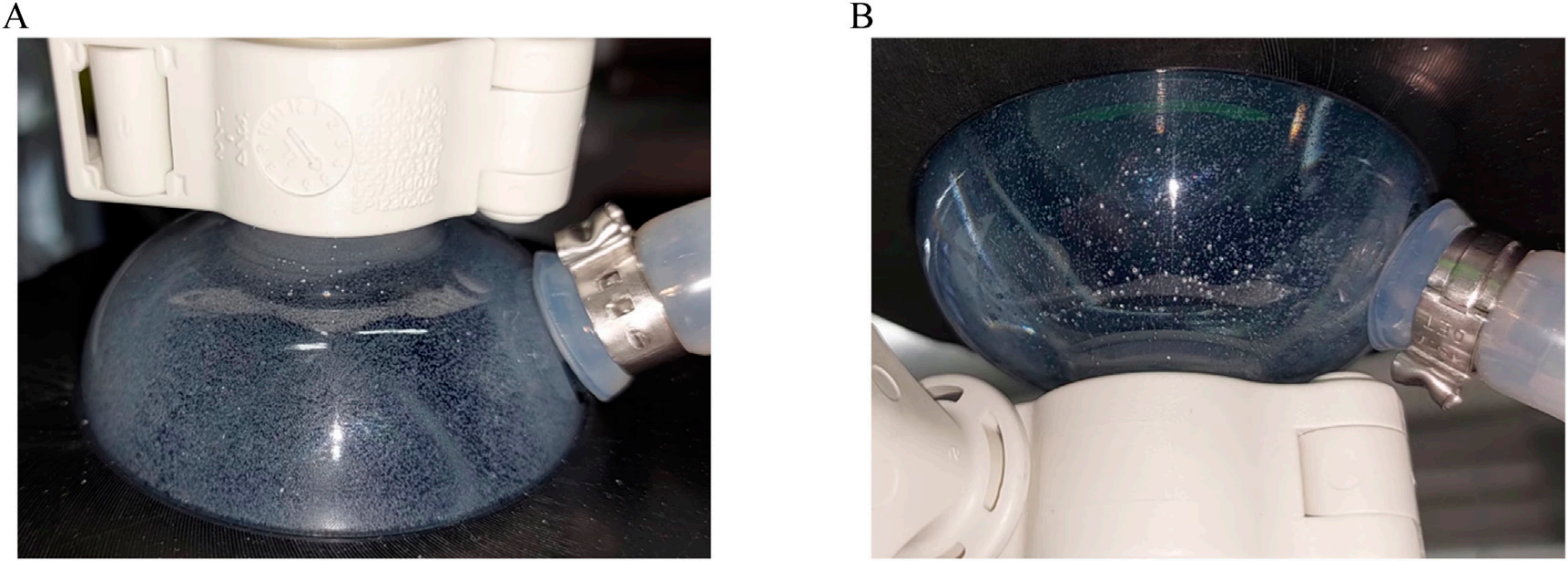
Diaphragm pump of ATF-2 device installed upright **(A)** and upside down **(B)**. Photographs were taken at the end of the pressurizing phase, showing MCs trapped between the pump housing and membrane.

With the ATF system, a flow rate of 0.5 L min^−1^ decreased the agitation rate required to reach *N*_*s1u*_ from 57 to 45 rpm. During the pressurizing phase of the ATF cycle, the bottom of the reactor was flushed by the high instantaneous out-flow, leading to a breakup of stationary MC deposits. However, during the exhaust phase, MC deposit formation occurred. For this reason and to ensure comparability between the cultivations, the same agitation rate of 57 rpm was used for the ATF cultivation. The installation orientation, regular or upside down, of the ATF-2 device was also investigated since sedimentation of MCs in the diaphragm pump was deemed potentially problematic. While it was noticeable that some MCs got trapped between the diaphragm and the pump wall (Figure 2), no accumulation over time was observed. While rotating the ATF-2 reduced the number of MCs stuck in the pump, it resulted in undesirable gas accumulation.

### Cell growth

**FIGURE 3:**
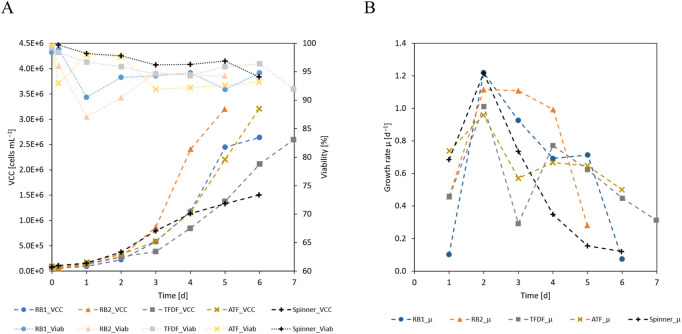
**(A)** VCC and viability and **(B)** cell specific growth rate of the cultivations.

The first sample was taken after 24 h of cultivation, at the end of the intermitted agitation attachment phase, and the number of attached cells was measured by trypsinization of the MCs. The 24 h attachment efficiency was calculated by forming the ratio between the measured VCD and the seeding VCD. Since the cells only demonstrated a short lag-phase, proliferation over the first cultivation day resulted in 24 h attachment values in excess of 100%. Specifically, this figure ranged from 121% for the RB1 cultivation to 232% for the ATF cultivation while it was 201% in the spinner flask. As shown in Figure 3B, the cell specific growth rates (*µ*) peaked at values of ≈1 day^−1^ on day 2 before they started to fall at different rates, depending on the cultivation. On day 2 of the cultivations, perfusion was started, leading to diverging cellular growth rates. Especially in the TFDF cultivation, a severe drop of 70% was noticeable. As shown in Figure 3A, viable cell concentrations (VCCs) increased with the cultivation duration and maximum values of 2.64 · 10^6^ cells mL^−1^ and 3.20 · 10^6^ cells mL^−1^ were reached in RB1 and RB2, respectively, while the perfusion cultivations using the ATF or TFDF cell retention devices reached maximum VCCs of 3.21 · 10^6^ cells mL^−1^ and 2.60 · 10^6^ cells mL^−1^, respectively. In the spinner flask comparison cultivations, maximal VCCs of ≈1.50 · 10^6^ cells mL^−1^ were reached. Due to differences in growth rates, the cultivation duration required to reach the maximal VCC varied from 5 to 7 days. During the entire expansion, cell viability dropped from 99% to values between 90%–95%. However, it should be mentioned that the observed decrease in viability could at least partly be caused by sample handling, as prolonged exposure to TrypLE and strong agitation were seen to have resulted in decreased values.

### Cell metabolism

**FIGURE 4:**
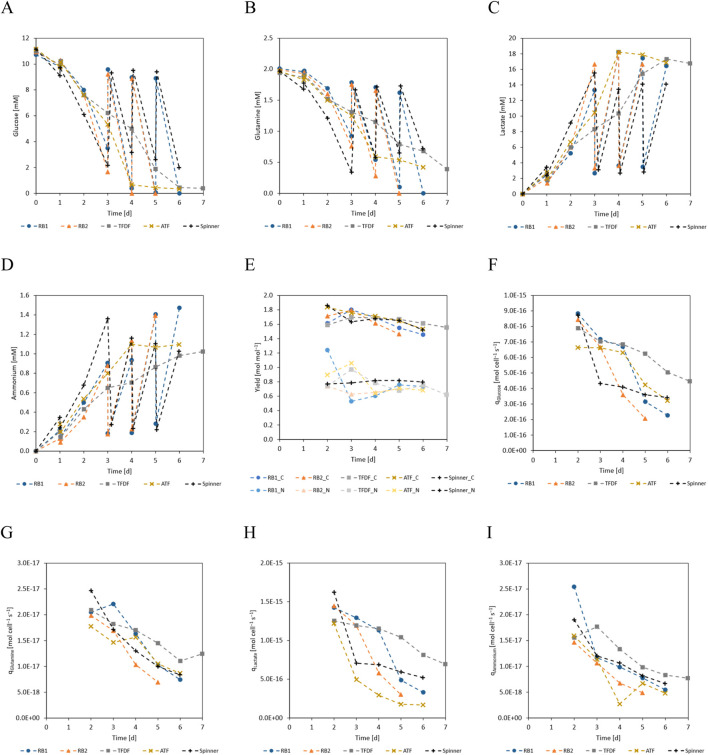
**(A)** Glucose, **(B)** glutamine, **(C)** lactate, **(D)** ammonium concentrations over cultivation time. Glutamine concentration represents the sum of free glutamine and alanyl-glutamine. **(E)** Lactate yield from glucose (C-metabolism) and ammonium yield from glutamine (N-metabolism). **(F)** Cell specific glucose consumption rate. **(G)** Cell specific glutamine consumption rate. **(H)** Cell specific lactate production rate. **(I)** Cell specific ammonium production rate. Because the concentration changes during the first 24 h were small compared to inherent inaccuracies of the analytical methods, the yields and cell specific production/consumption are not displayed for this period.

One main difference between the repeated-batch and the perfusion processes was the progression of the substrates and metabolites concentrations. As visualized in Figure 4, the daily MEs of the repeated-batch cultivations led to step like concentration changes while this was smoothed out by the perfusion operation mode. Furthermore, glucose and glutamine were periodically depleted in the repeated-batch cultivations from cultivation day 4 onward. In the last 2 days of the perfusion cultivations, the glucose concentration fell below 0.5 mM, indicating potential substrate limitations. Contrary to the repeated-batch cultivations, glutamine was never depleted in the perfusion cultivations and its concentration only dropped to ≈0.4 mM on the last cultivation day. In conjunction with the lower cell densities of the spinner cultivation, less substrate was consumed and no indication for substrate limitations was found. The lactate yield from glucose (Figure 4E) decreased slightly with culture progression while no apparent trend was visible in the ammonium from glutamine yield. In general, no substantial differences between the yields of the different cultivation modes could be found. The cell specific production and consumption rates of the measured substrates and metabolites (Figures 4F–I) followed a similar trend as the cell specific growth rate and decreased with time. While the curves were similar for all cultures, the TFDF cultivations tended to have the highest values.

**FIGURE 5:**
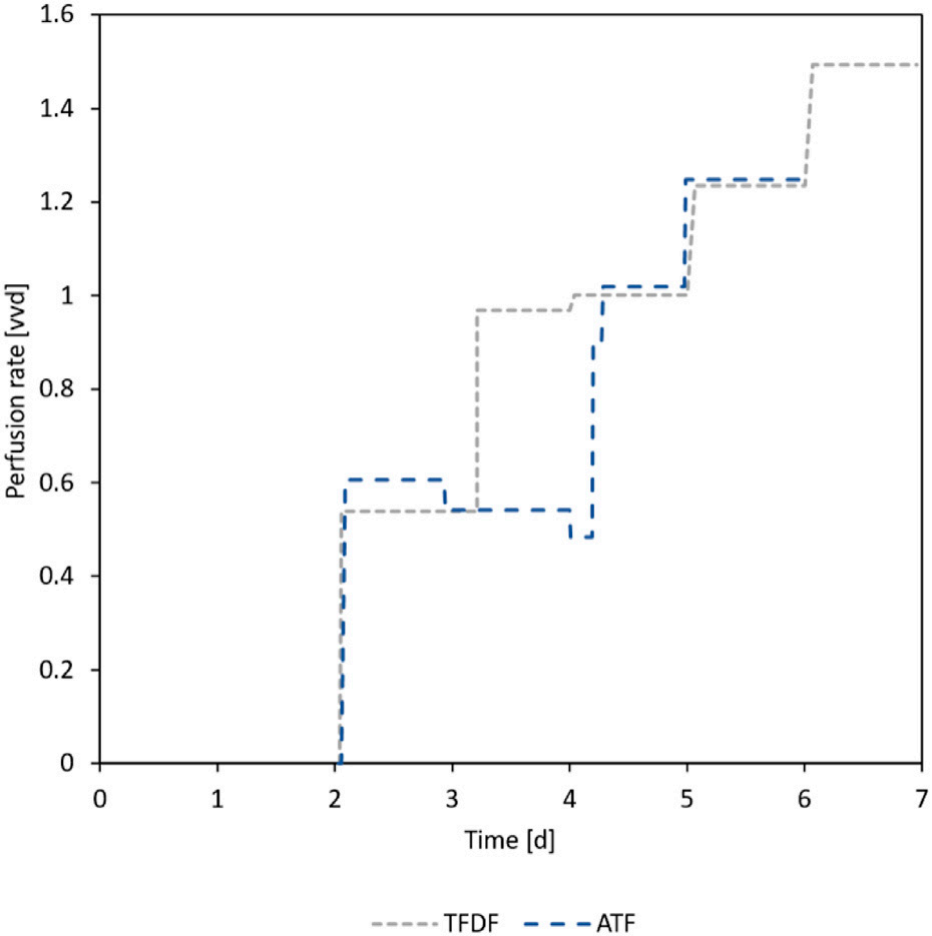
Applied perfusion rates during ATF and TFDF cultivation. On day 2, the respective perfusion devices were primed, and perfusion operation was started.

As can be seen in Figure 4A, the trend of glucose concentration in the first 3 days of the repeated-batch cultivations indicated an imminent substrate shortage, necessitating the start of the daily MEs. In contrast to the repeated-batch, the addition of new substrates in the perfusion mode is gradual and can be said to lag behind the instantaneous partial MEs. For this reason, it was decided to start the perfusion operation already on day 2 of the cultivation at a perfusion rate of 0.5 vvd. Based on the course of the substrate concentrations, the perfusion rate was gradually increased, as can be seen in Figure 5. In total, including the 1.8 L required to initially fill the bioreactor, 10.2 L medium was used in the TFDF cultivation while 7.2 L was used in the ATF cultivation. The medium consumption of the RB1 and RB2 cultivations was lower at 6.1 L and 4.7 L, respectively.

### Aggregation behavior and cell distribution

**FIGURE 6:**
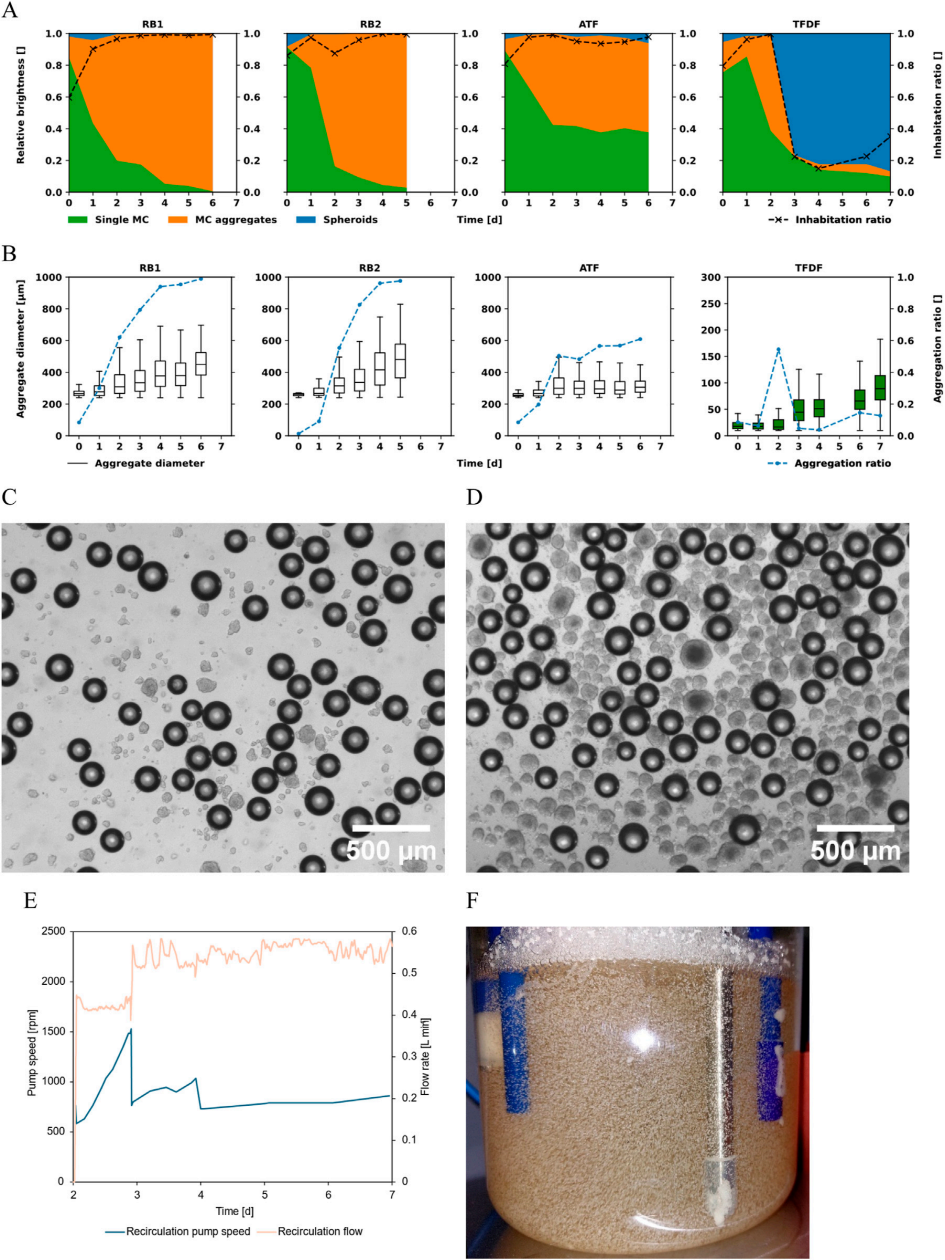
**(A)** Relative prevalence of cells on single MCs, aggregated MCs, and spheroids. The inhabitation ratio corresponds to the percentage of MCs showing at least 1 cell. **(B)** Size distribution of MC aggregates and aggregation ratio over time. For the TFDF cultivation, the boxplots show the spheroid diameters (green) instead of the MC aggregate diameters (white). A threshold of 240 µm was chosen to differentiate single MCs from MC aggregates based on diameter. Microscope images of samples taken from the TFDF cultivation on day 3 **(C)** and day 7 **(D)**. The darker spheres are MCs while the spheroids are of lighter color. **(E)** Recirculation pump speed and flow during the TFDF cultivation. On day 3 and 4, the circulation loop was re-primed. **(F)** Picture of RB2 cultivation on harvest day. Visible are large MC aggregates stuck between reactor internals and wall.

To analyze cell distribution and MC aggregation, samples were stained with DAPI and evaluated using a fluorescence microscope. The area of bright regions in the DAPI channel (cells) and dark regions in the brightfield channel (MCs) was assessed by counting the respective pixels in the recorded pictures. The bright pixels detected in the DAPI channel were attributed to the specific MC they fell on. Regions which showed DAPI signals unassociated with MCs were classified as spheroids. Single MCs and MC aggregates were distinguished based on the projected area they covered such that two or more MCs bridged together by cells were classified as an aggregate, the same definition has also been used by others (Jossen et al., 2016). All detected bright pixels in the DAPI channel were thusly sorted into one of these three categories. The relative brightness, calculated by dividing the number of bright pixels in each category by the total number of bright pixels, is displayed in Figure 6A. The concurrently displayed inhabitation ratio corresponds to the percentage of MCs that had at least one bright DAPI region associated with them. After the initial attachment phase of 24 h, cells were detectable on more than 90% of the analyzed MCs in all cultivations. A major difference between the cultivations was the aggregation behavior of the MCs, as can be seen in Figure 6B. While the median MC aggregate diameter rose to 470 µm (≈10 MCs per aggregate) in the repeated-batch cultivations, it remained at ≈250 µm (≈2.5 MCs per aggregate) in the ATF cultivation. Additionally, more than 95% of the MCs were incorporated in aggregates by the end of the repeated-batch cultivations while the aggregation ratio stabilized at ≈60% in the ATF cultivation. Up to day 2, the TFDF cultivation showed the same trend as the other cultivations. However, with the start of the recirculation loop, most of the cells were stripped from the MCs by the high shear forces present in the recirculation pump. This could be seen as a strong increase in the spheroids fraction (Figure 6A). Only ≈20% of the detected cells were still associated with MCs at day 3 of the cultivation. In all other cultivations, the fraction of cells not associated with MCs remained negligible. The median diameter of the spheroids present in the TFDF cultivation rose to ≈90 µm. Images of the MCs and spheroids of the TFDF cultivation are shown in Figures 6C,D. Especially in the first 2 days of the perfusion operation, gas buildup was observed in the recirculation loop of the TFDF cultivation. To keep the recirculation flow rate at its setpoint, the system increased the impeller speed of the pump, as can be seen in Figure 6E. It was likely this increase in impeller speed that led to the stripping of the cells from the MCs. Once this issue was discovered, the flow path was re-primed, and the recirculation flow rate was increased to 0.5 L min^−1^. Between day 3 and 4, gas buildup again led to an increase in impeller speed, necessitating re-priming the recirculation loop. Subsequently, no further gas accumulation was observed.

### Harvest and cell quality

During the harvest of the perfusion bioreactor runs, the applicability of their respective cell retention devices for cell washing was assessed. The aim was to carry out a volume reduction and subsequent diafiltration with PBS to remove the cultivation medium before the enzymatic cell dissociation reagent was added. In the case of the TFDF cultivation, this strategy had to be abandoned as the filter quickly became clogged. The ATF proved to be more promising, however, the permeate flux had to be limited to 40 mL min^−1^ to prevent excessive transmembrane pressures and filter fouling.

The key data of the harvested bioreactors is listed in Table 4. The viability after the harvest and MC filtration step was >95% in all cultivations, indicating that cells were not damaged during the process. The harvest efficiency, which indicates percentage of cells that could be recovered post-harvest when compared to the expected cell yield based on the last sampling prior to harvest, ranged from 42.1% to 85.4% for the MC based cultivations. In case of the TFDF cultivation, which was mainly comprised of spheroids at the time of harvest, a higher harvest efficiency of 103% was achieved. After cryogenic storage, the quality of the harvested cells was assessed by flowcytometry and directed differentiation assays.

**TABLE 4:** Key harvest data after MC filtration step.

Run	Max VCD [cells cm^−2^]	Max VCC [cells mL^−1^]	Process duration [d]	Expansion factor [ ]	Average *µ* [d^−1^]	Population doubling level [ ]	Harvest efficiency [%]	Viability post-harvest [%]	Cell yield [cells]
RB1	7.34 · 10^5^	2.64 · 10^6^	6	44.7	0.633	5.48	74.1	96.5	3.33 · 10^9^
RB2	8.89 · 10^5^	3.20 · 10^6^	5	56.7	0.808	5.83	42.1	95.4	2.29 · 10^9^
TFDF	7.21 · 10^5^	2.60 · 10^6^	7	40.7	0.531	5.78	103	99.2	4.54 · 10^9^
ATF	8.91 · 10^5^	3.21 · 10^6^	6	54.9	0.669	5.35	85.4	97.3	4.66 · 10^9^

Based on the flow cytometric assessment of the harvested cells, no difference in cell quality could be detected between the different expansion processes. Interpretation of flowcytometry data was based on the position paper by Dominici et al., which states that positive markers should be expressed by > 95% of all cells, while the negative marker expression should be <2% (Dominici et al., 2006). As can be seen in Table 5, these criteria were fulfilled in all cultivations and >97% of the cell population demonstrated the complete surface marker profile attributed to hMSCs.

**TABLE 5:** Marker expression of hMSCs after expansion in the BioBLU 3c bioreactor. The hMSC^+^ figure indicates the percentage of the population that displayed all positive and none of the negative markers.

Cultivation	Neg. Markers [%]	CD73 [%]	CD90 [%]	CD105 [%]	hMSC^+^ [%]
RB1	1.6	98.7	98.8	99.2	97.8
RB2	1.6	98.8	98.8	99.0	97.6
TFDF	1.4	99.9	99.9	100	98.6
ATF	1.9	99.8	99.8	99.8	97.9

**FIGURE 7:**
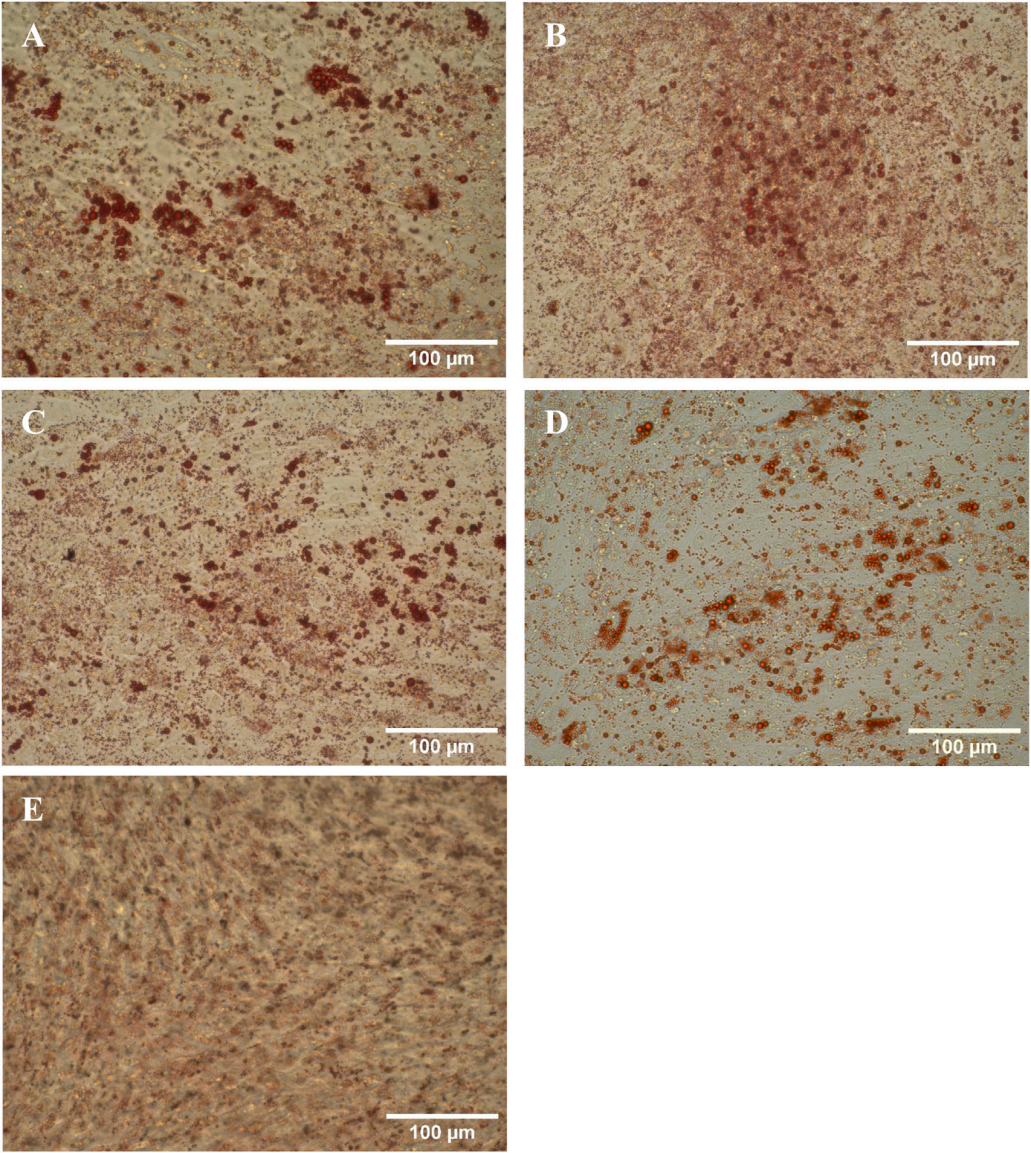
Harvested hMSCs differentiated into adipocytes and stained with Oil Red O. **(A)** RB1, **(B)** RB2, **(C)** TFDF, **(D)** ATF, **(E)** negative control.

The directed differentiation of the harvested cells into adipocytes was successful as the presence of oil droplets stained by Oil Red O can be clearly seen in Figure 7. While there were also a few oil droplets present in the negative control, they were not as large and numerous.

**FIGURE 8:**
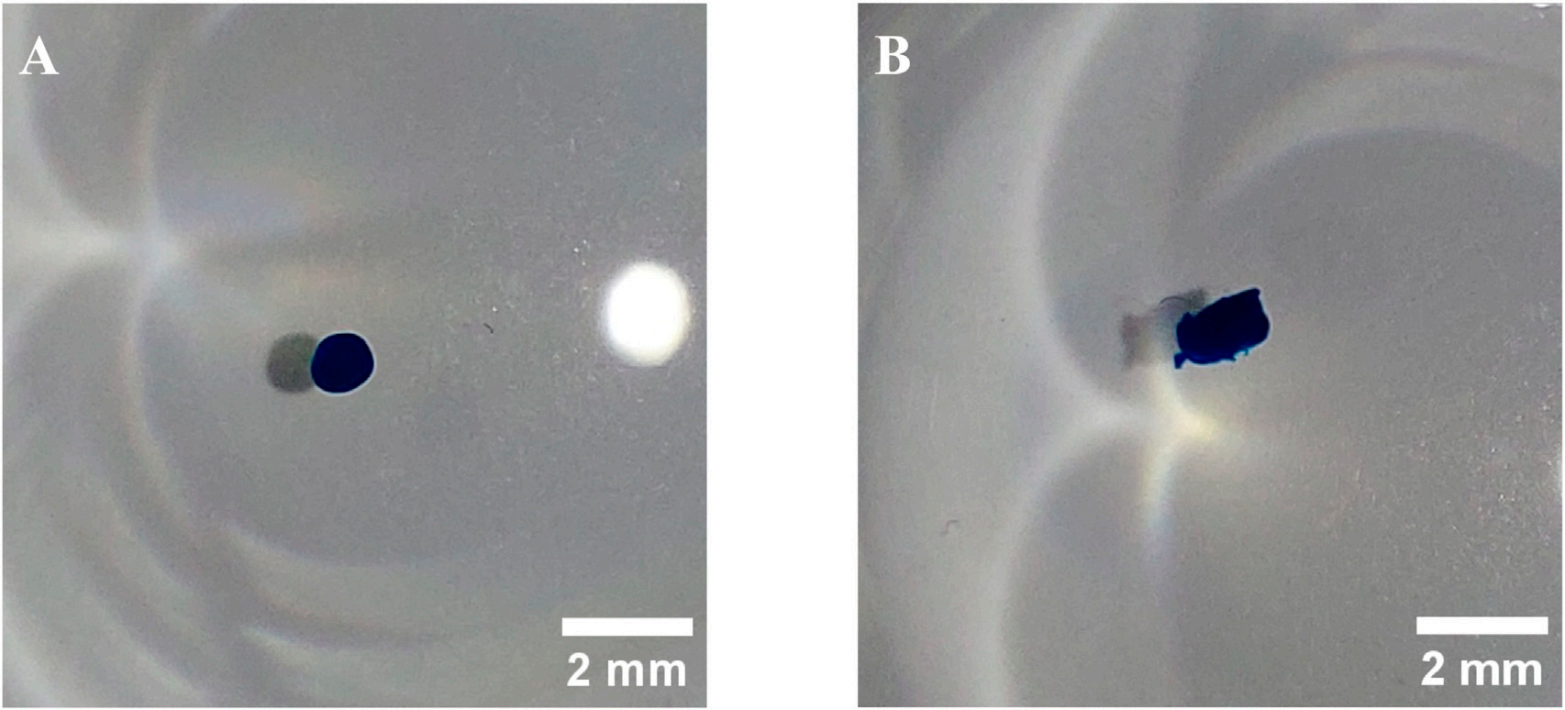
Examples of hMSCs differentiated into chondrocytes and stained with Alcian blue. **(A)** RB2, **(B)** negative control.

The success of the chondrogenic differentiation was assessed by alcian blue staining. After 21 days cultivation in the differentiation medium, hard cartilage like spheroids were formed. After fixation, the spheroids were stained and assessed optically. In all cases, a deep blue coloration was present, and an example is depicted in Figure 8A. However, the negative control, a pellet of undifferentiated cells, was also stained to a similar degree, calling the validity of the used staining method into question. Nevertheless, the morphology and consistency of the generated chondrocyte spheroids differed strongly from the negative control, suggesting successful differentiation.

**FIGURE 9:**
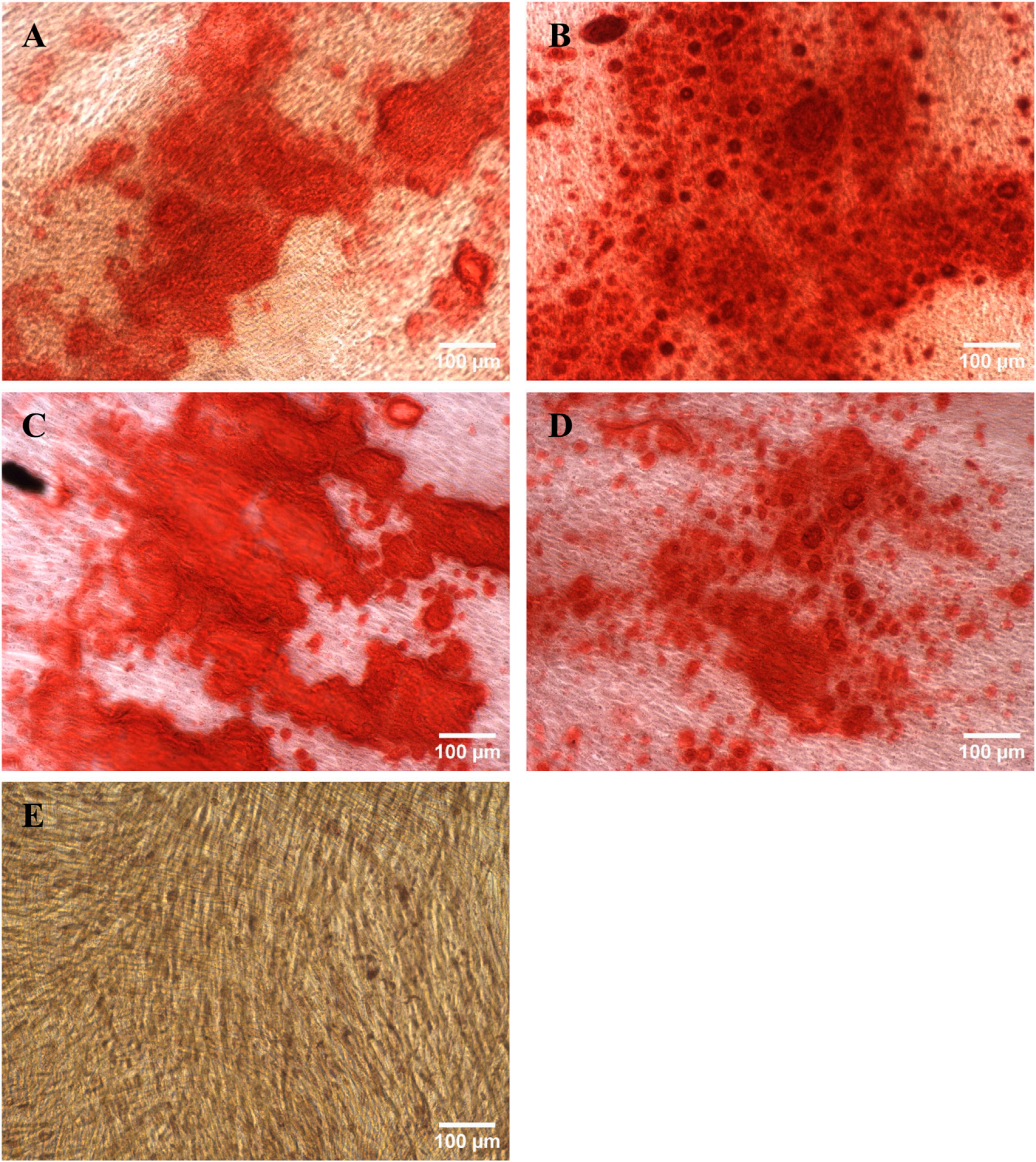
Harvested hMSCs differentiated into osteoblasts and stained with Alizarin Red S. **(A)** RB1, **(B)** RB2, **(C)** TFDF, **(D)** ATF, **(E)** negative control.

The osteogenic differentiation potential was confirmed by Alizarin Red S staining. As can be seen in Figure 9, large areas of calcium deposits where stained red in the differentiated cell samples while the negative control remained colorless.

## Discussion

Since hMSCs are thought of as shear sensitive, the mechanical and hydrodynamic stress caused by the stirrer must be minimized (Nogueira, Cabral, and Rodrigues, 2021). For this reason, the agitation rate was set to *N*_*s1u*_ as this minimizes shear stress while ensuring that no stationary MC deposits are formed (Schirmaier et al., 2014). However, the utilized cell retention devices also impart their own fluid flow, resulting in an increased power input. While this leads to higher shear stress, it may also assist the MC suspension and conversely reduce the agitation rate required to reach *N*_*s1u*_, as it was found to be the case with the ATF system. While the power input of the TFDF system did not noticeably influence the *N*_*s1u*_, it evidently increased the shear stress experienced by the cells to detrimental levels. Additionally, a large amount of thermal energy was lost through the exposed surface of the TFDF flow path, requiring the bioreactor controller to continuously increase its heating output from 20% to 80%.

Cell densities reached in the BioBLU 3c STRs were at least twice as high as in the spinner flask control cultivations, indicating that the pH and DO controlled environment strongly improved cell growth. For the first two cultivation days, all bioreactors were treated identically. Nevertheless, variations were present on day 2 of cultivation, showcasing the natural variability of the cultivated cells. The decreased cell proliferation in the TFDF cultivation was mainly due to the cell detachment caused by the recirculation pump. While VCD continued increasing after the start of the cell retention device, it did so in a reduced, linear fashion. This was better than expected, as a quick stagnation of hMSC spheroid culture growth is described in literature (Barekzai et al., 2023; Petry and Denise, 2022). To some degree, the decreased growth rate might also be an effect of the high shear stress experienced during forceful detachment (Tower, 2012; Jossen et al., 2016). The decline in growth rate observed in the other cultivations was most likely due to growth area limitations, as the MCs were fully confluent at the end of the cultivation, leading to contact inhibition (Marescal and Cheeseman, 2020).

On the last 2 days of the repeated-batch cultivations, glucose and glutamine depletions were observed, meaning that the 80% MEs performed daily were not enough to ensure the nutrient supply of cells. While it is possible to carry out 100% MEs, it is disproportionally more challenging in MC cultivations since the dip-tube for the medium removal needs to reach all the way to the bottom of the reactor, increasing the risks of MC loss or blockage of the MC retention dip-tube. Another option to increase the nutrient supply is performing multiple MEs per day, however, this comes with vastly increased operator workload. These challenges can be circumvented by switching to perfusion operation mode, as this enables adapting the medium inflow based on the culture’s substrate consumption. Still, during the last 2 days of the perfusion cultivations, the glucose concentration fell below 0.5 mM, suggesting that substrate limitation may have occurred. However, the continuous renewal of the medium through the perfusion ensured that the cells were never completely without substrates, alleviating the potential negative effects of the substrate limitation. The decrease of the lactate from glucose yield towards the end of the cultivations could also partially be caused by glucose limitation, forcing the cells increase energy production through oxidative phosphorylation and therefore decreasing lactate production. In no cultivation did the metabolites lactate and ammonium exceed concentrations of 18 mM and 1.4 mM, respectively, suggesting that no inhibitory effects due to metabolite accumulation were present, as these effects are not expected below concentrations of 36 mM for lactate and 2.4 mM for ammonium (Pattappa et al., 2011; Schop et al., 2009).

Despite the similar maximum cell densities reached, the medium usage of the four cultivations differed substantially. While the daily ME of the repeated-batch cultivations corresponded to a dilution rate of 0.8 vvd, exchange rates of up to 1.5 vvd were applied in the perfusion cultivation. This, combined with the longer cultivation duration, resulted in the TFDF cultivation using more than twice as much medium as the RB2 cultivation. Since the medium is one of the main cost drivers, this is problematic. However, by adapting the feeding strategy and potentially developing specialized perfusion media, it should be possible to reduce the medium consumption and the associated costs to levels similar to the repeated-batch process.

In MC cultivations, the size distribution of the formed aggregates is a critical process attribute since nutrient and oxygen limitations can be present in the aggregates cores, which, in extreme cases, can lead to cell death (Kinney, Sargent, and McDevitt, 2011). Specifically, to avoid diffusion-based limitations, the cell layer should be kept below a thickness of 150 µm (Wu et al., 2014). While the median MC aggregate diameter rose to ≈450 µm in the repeated-batch cultivations, the thickness of the cell layer itself was still below the critical threshold in most cases. This was due to the spongy nature of the MC aggregates, allowing medium to flow through the openings between the MCs. The aggregate formation in the repeated-batch cultivations was likely exacerbated by the ME procedure as it required prior sedimentation of the MCs to avoid clogging of the MC retention dip-tube. During this, the MCs and cells were in close contact for at least 15 min, resulting in the formation of large MC aggregates with diameters of up to 20 mm. While these large aggregates were dispersed by the stirrer over time, the aggregation could not be reversed completely.

In the ATF cultivation, the formation of large MC aggregates was almost entirely suppressed. This was most likely due to the mechanical forces the MC aggregates experienced when they were pushed through the lumen of the hollow fiber filter. As these have an inner diameter of 1 mm, any aggregate larger than this would have been torn apart. These smaller and more uniformly sized MC aggregates should result in improved oxygen and nutrient supply to the hMSCs due to the reduced diffusion distances (Wu et al., 2014).

Although the PuraLev pump integrated in the TFDF recirculation loop is considered to be a low-shear pump suitable for mammalian cell cultivation (Dittler et al., 2014), the imparted forces resulted in the cells being stripped from the MCs. Remarkably, this did not result in cell death but instead transformed the MC cultivation into a spheroid cultivation with the cells still proliferating, albeit at a reduced growth rate. The gas buildup that led to the increased pump impeller speeds was likely caused by CO_2_ outgassing, as it decreased together with the output of the pH-controlled CO_2_ mass flow controller. Direct introduction of bubbles through sparging is considered unlikely since the input dip-tube of the TFDF loop was not located close to the sparger. At harvest, 75% of the spheroids had diameters of <110 μm, however, a small number with diameters >300 µm were also present. While there is literature describing the cultivation of stem cells as spheroids, it mainly focuses on induced pluripotent stem cells (Vallabhaneni et al., 2023; Abecasis et al., 2017). The available studies on hMSC spheroid cultivations describe methods with limited scalability that are mainly intended for small-scale biological studies and not for large-scale biomass generation (Ryu, Lee, and Park, 2019; Egger et al., 2018). Therefore, the KrosFlo TFDF Lab System may show merit for the initiation and expansion of hMSC spheroid cultures. However, it needs to be confirmed that this also works with primary hMSCs.

Currently, harvesting and downstream processing is still a bottle neck in large stem cell production as gentle washing procedures have to be performed in a timely manner (Cunha et al., 2015b). While washing of the MCs prior to harvest was not possible with the TFDF system, a diafiltration step could be successfully performed using the ATF system. However, during the volume reduction and diafiltration step, filter fouling was observed which necessitated lowering the permeate flow rate, resulting in this step taking approximately 1 h. While a processing time in this range should not negatively affect cell quality, shortening it would be beneficial, also from a labor expense standpoint. One option to circumvent filter fouling in both systems would be to increase the tangential flow rate. However, this would also increase the hydrodynamic stress, which could negatively affect cell quality. A better option is to use hollow fiber filters with larger pore sizes. The standard filter configuration of the ATF-2 single use device consists of 1,300 cm^2^ filter area with a pore size of 0.2 µm, which is much smaller than what is necessary to retain the cells in the bioreactor. In the case of MC cultivation, it would actually be feasible to use pore sizes of up to 100 µm since the cells are bound to the much larger MCs. While the depth filter used in the TFDF cultivation featured larger pores of 3–5 μm, its much smaller surface area of 30 cm^2^ was probably the reason for the quicker clogging.

In MC based hMSC cultivations, the harvest process including cell washing and MC separation is reported to have an efficiency of around 80% (Borys et al., 2021), which similar to what has been achieved in this study, with the exception of the RB2 cultivation with a harvest efficiency of only 42.1%. A reason for this could be the excessive aggregation present at the end of this cultivation as the harvesting enzyme may not have been able to penetrate and disperse the large aggregates (Rafiq et al., 2017). This is supported by the fact that of the MC based cultivations, the ATF cultivation, which had no large aggregates, achieved the highest harvest efficiency. Potentially, harvest efficiency could have been improved by applying short bursts of strong agitation. It has been described in literature that harvest efficiencies >95% may be achieved in this manner without adversely affecting the hMSC quality (Nienow et al., 2014). Curiously, the harvest efficiency of the TFDF cultivation, which consisted mostly of spheroids at the time of harvest, was very close to the ideal value of 100%, indicating that spheroids with diameters of up to 200 µm could be successfully dispersed through the applied harvesting procedure. However, it should be noted that the observed harvest efficiencies are directly dependent on the last VCD measurement prior to the harvest. Strong aggregation can influence the sedimentation behavior of the MCs, leading to an inhomogeneous distribution within the bioreactor and therefore to biased sampling. The inconsistent presence or absence of large aggregates in the relatively small sampling volume used to determine VCD additionally increased measurement uncertainty. The lower harvest efficiencies of the MC cultivations could therefore indicate that the VCDs measured during the cultivation where overestimated compared to the TFDF cultivation. This could explain the slightly higher calculated cell specific substrate consumption and metabolite production rates of the TFDF cultivation.

Based on flowcytometric surface marker assessment and the differentiation assays, no difference in cell quality could be discerned between the inoculum and the harvests from the different bioreactor cultivations. Additionally, the viability of the cells after harvest was substantially higher than the 70% stated as the minimum acceptable level by the U.S. Food and Drug Administration (Chen et al., 2013). However, the largest loss of viability is typically only observed after cryopreservation (Moll et al., 2014). Assessing the chondrogenic differentiation potential however proved to be difficult. Different alcian blue dye concentrations, ranging from 1 to 10 g L^−1^, dissolved in various combinations of ethanol, acetic acid and water at pH between one and 2.5 were tested. While the staining intensity differed with the chosen staining solution and duration, it was not possible to clearly distinguish between negative and positive samples based on color. It was also found that fixation influenced the staining intensity with 10% formalin producing a stronger color than Saccomanno’s fixative. Destaining, even with 1 M HCl was found to not be effective. Through cutting a differentiated chondrocyte nodule after staining it was found that the dye was not able to penetrate the hard tissue and mostly the outer cell layer was stained. Based on our observations, it appears that alcian blue staining of whole chondrocyte nodules is not sufficiently selective, stressing the importance of negative controls, which are not shown in many of the publications cited in this study (da Silva et al., 2019; Dos Santos et al., 2014; Lawson et al., 2017; Cunha et al., 2017). Nevertheless, it is likely that the difference between the differentiated sample and the negative control would have been clearer if microtome sections were stained instead of whole spheroids (Vemuri, Chase, and Rao, 2011). The differentiation capabilities of hMSCs were originally defined as an identifying criteria by Dominici et al. (2006) but are not necessarily related to their therapeutic potency as more recent literature suggests that their beneficial properties are mainly conveyed through cell signaling and production of extracellular vesicles (Mushahary et al., 2018; Aldahmash et al., 2012; Bagno et al., 2022). Immune assays, such as T cell suppression assays or tumor necrosis factor-alpha expression in MSC co-culture, might be more informative as they asses the immunomodulatory potency of the hMSCs and may also be applicable for assessing the effects of produced extracellular vesicles (Robb et al., 2019; Galipeau et al., 2016).

## Conclusion

In summary, the applicability of ATF for MC based hMSC cultivations could be shown while the working principle of the TFDF system might be more suitable for spheroid cultivations. While the achieved maximum VCDs were not higher in the perfusion cultivations than in the repeated-batch cultivations, the full potential of perfusion was not utilized in this proof-of-concept study as the growth surface to medium ratios were kept identical in all cultivations. The automated medium replenishment offered by perfusion makes it possible to utilize much higher MC concentrations, resulting in increased space-time yields. Even without this optimization step, perfusion mode saved approximately 30 min of labor time each day by eliminating manual MEs, lowering the labor costs and contamination risk. Additionally, the application of cell retention devices for washing during harvesting shows merit. While filter blockage proved to be a limiting factor, this can be alleviated by larger pore sizes or filter areas. Ultimately, at the larger scales required for the cost-efficient production of allogenic hMSC therapies, the washing steps and buffer exchanges required during harvest will only grow more challenging (Tsai and Pacak, 2021), making cell retention devices a necessity. Furthermore, the use of perfusion devices has to be evaluated for the production of extracellular vesicle, as cell-free extra cellular vesicles could be harvested continuously and in a scalable manner (Ulpiano et al., 2025).

## Data Availability

The original contributions presented in the study are included in the article/supplementary material, further inquiries can be directed to the corresponding author.

## Abbreviations

AQ, Alanyl-glutamine; ATF, Alternating tangential flow filtration; DAPI, 4′,6-diamidino-2-phenylindole; DO, Dissolved oxygen; EDTA, Ethylenediaminetetraacetic acid; Glc, Glucose; Gln, Glutamine; hMSC, Human mesenchymal stromal cell; Lac, Lactate; MC, Microcarrier; ME, Medium exchange; NH_4_^+^, Ammonium; *N*_*s1u*_, Lower limit of Zwietering’s suspension criterion ; PBS, Phosphate buffered saline; RB, Repeated batch; STR, Stirred tank reactor; TCD, Total cell density; TFDF, Tangential flow depth filtration; *µ*, Cell specific growth rate; VCD, Viable cell density; VCC, Viable cell concentration; vvd, Vessel volume per day.
